# Identification of SNPs in candidate genes related to water stress in *Eucalyptus*

**DOI:** 10.1186/1753-6561-5-S7-P52

**Published:** 2011-09-13

**Authors:** Cíntia Helena Duarte Sagawa, Leonardo Curi Martin, Andréia Santoro, Julio Cezar Santos Otto, Juliana Erika de Carvalho Teixeira, Rinal do César de Paula, Celso Luis Marino

**Affiliations:** 1 Departamento de Genética, UNESP, Botucatu, São Paulo, 18618-970, Brazil; 2 FIBRIA, São Paulo, 12340-010, Brazil; 3 Departamento de Produção Vegetal, UNESP, Jaboticabal, São Paulo, 14884-900, Brazil

## Background

Forestry companies have occupied Northern and Northeastern of Brazil with plantations of *Eucalyptus* species; however, limitations on water supply are affecting the biomass production and reducing the yield significantly [1].Tolerant plants develop defense mechanisms like hormone production of abscisic acid (ABA) and osmoprotector glycine betain (GB) when submitted to drought conditions [2]. Identifying and studying genomic regions related to water stress tolerance are important for tree improvement programs. In this work, SNPs in candidate genes, 9-cis-epoxy-carotenoid dioxygenase (NCED) and choline monooxygenase (CMO), of ABA and GB biosynthetic pathways related to drought tolerance in a population of *Eucalyptus* were identified, as well as, a possible molecular marker associated to genomic regions related to plant response to water stress through AFLP technique [3] combined with bulk segregant analysis method [4].

## Materials and methods

Contrasting plants of *E. grandis*, *E. urophylla,* and their hybrids were selected according to their physiology. Specific primers were designed from homology sequences from *Eucalyptus* ESTs databank and amplification products submitted to sequencing which allowed the identification of SNPs and the genotyping of these plants. For the identification of the AFLP marker, contrasting DNA bulks were digested with restriction enzymes combination and routine protocols were followed. The selective amplification products were separated on 6% denaturing polyacrylamide gel and seen in the silver nitrate staining [5].

## Results and conclusions

Seven SNPs were identified in a region of 1230 bp on NCED gene from which five were in codified regions and generated synonymous mutations. For the CMO gene, 49 SNP were identified in a region of 3885 bp, which 12 were in codified regions and 37 in UTRs and intron regions. Especially for these codified regions; 83,3% of the mutations were synonymous and 16,7% were non-synonymous. Through the genotyping of the SNPs, the NCED and CMO genes presented respectively, seven haplotypes with 15 different genotypes and 18 haplotypes with 16 diverse combinations. Nevertheless, CMO gene showed some unique genotypes for some species. Then, the genotyping of individuals by the allele-specific extension technique demonstrated to be efficient; moreover the SNPs primers designed can decrease costs and permit the genotyping of these mutations in large scale of contrasting populations to water deficit and in population studies. From the analysis of the DNA bulks, 50 AFLP primer combinations were tested from which 27 generated polymorphic bands between the bulks and just one primer combination was confirmed in all susceptible plants of the bulk by now. This fragment sequence will be compared and converted into PCR-based markers.
